# Management of Acute Wilsonian Hepatitis with Severe Hemolysis: A Successful Combination of Chelation and MARS Dialysis

**DOI:** 10.1155/2021/5583654

**Published:** 2021-05-11

**Authors:** Jeremy Hassoun, Nathalie Hammer, Giulia Magini, Belen Ponte, Marie Ongaro, Anne-Laure Rougemont, Nicolas Goossens, Jean-Louis Frossard, Laurent Spahr

**Affiliations:** ^1^Gastroenterology and Hepatology, University Hospitals of Geneva and Faculty of Medicine, Geneva, Switzerland; ^2^Nephrology, University Hospitals of Geneva and Faculty of Medicine, Geneva, Switzerland; ^3^Clinical Pathology, University Hospitals of Geneva and Faculty of Medicine, Geneva, Switzerland

## Abstract

Wilson's disease is a rare hereditary disorder of copper metabolism leading to progressive accumulation of copper in several organs including the brain and the liver. Acute liver failure is a relatively rare hepatic manifestation of WD which may require urgent liver transplantation if medical treatment fails. We report here the case of a young woman who presented with classic acute Wilsonian hepatitis complicated by liver and renal failure and a severe hemolysis related to massive nonceruloplasmin bound copper accumulation requiring repeated blood transfusions. The early initiation of a combined treatment including conventional chelation therapy and repeated MARS dialysis sessions allowed a rapid control of hemolysis, a progressive decrease of free copper overload, and clinical recompensation without liver transplantation.

## 1. Introduction

Acute liver failure is a rare hepatic manifestation of WD [1], which may require urgent liver transplantation (LT) if medical treatment fails [2]. We report here the case of a young woman presenting with classic acute wilsonian hepatitis (AWH) with liver and renal failure and severe hemolysis related to massive nonceruloplasmin bound copper accumulation requiring repeated blood transfusions. The early initiation of a combined treatment including chelation therapy [3] and repeated MARS dialysis sessions allowed a rapid control of hemolysis, a progressive decrease of free copper overload, and a survival without urgent LT.

## 2. Case Presentation

**Figure 1:**
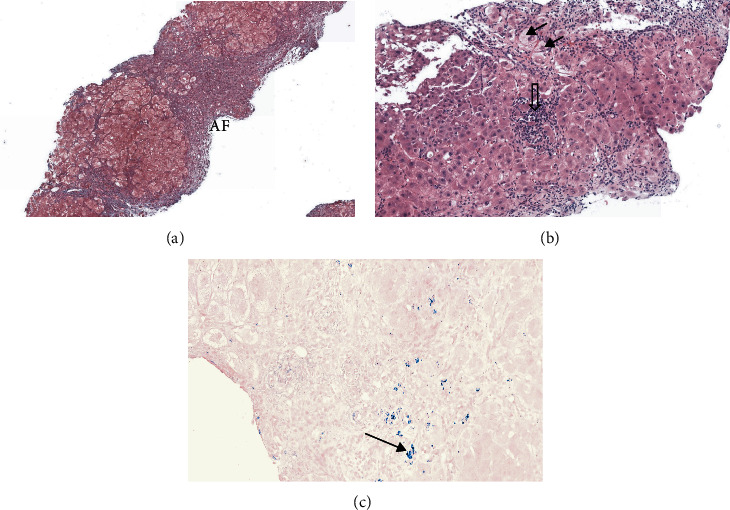
Liver biopsy specimen. (a) Annular fibrosis (AF) surrounding hepatocyte nodules (Masson trichrome staining, original magnification × 50). (b) Chronic hepatitis with lobular activity (open arrow), ballooned hepatocytes (black arrows), and mild steatosis (H&E staining, original magnification × 50). (c) Black arrows indicate Victoria blue staining deposits in periportal areas.

**Figure 2:**
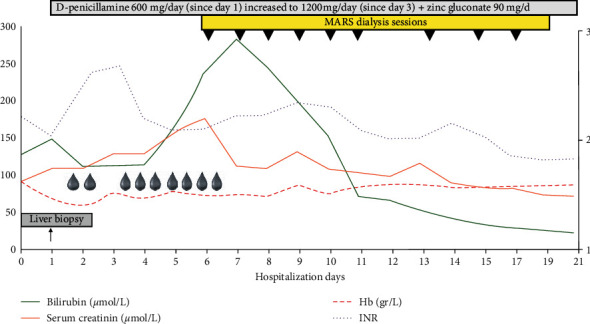
Graphical illustration of the patient's course during hospitalization. Each cartoon drop indicates a blood transfusion. The violet line indicates INR, and the red dotted line corresponds to hemoglobin level. The orange line and the green solid line illustrate serum creatinine and total bilirubin values, respectively. Black triangles correspond to the individual MARS dialysis session.

A 26-year-old female was admitted to our hospital following a 3-week history of anorexia, fatigue, and dark urines. She denied taking alcohol, recreative drugs, or any medications and had no family history of liver disease. On examination, except for jaundice, the patient had no signs of chronic liver disease, was alert, oriented, and afebrile. Blood pressure was 112/65 mmHg, and pulse rate was 88 beats per minute. At admission, blood tests showed mild AST elevation (3 times the upper limit of normal, ULN), normal ALT, low alkaline phosphatase (43 IU/L [N: >46], high gamma-glutamyltransferase (GGT) at 190 IU/L [N: <38], and elevated total bilirubin (128 *μ*mol/L [N: <2] with a conjugated form of 52 *μ*umol/L [N: 0.5–9.5]. Albumin level was 26 gr/l [N: 28–35]. Coagulation parameters were altered, with an elevated international normalized ratio (INR) of 2.34 and factor V 44% [N: >70]. Cell blood count demonstrated normal leucocytes and platelets, but low hemoglobin value of 67 gr/l [N: 120–160] with a high reticulocyte count of 293 G/L [N: 20–120)]. In the absence of overt bleeding, anemia was predominantly related to hemolysis with undetectable serum haptoglobin, high lactate dehydrogenase (LDH) [4], and negative Coomb's test. Further testing excluded hepatitis A, B, C, and E, as well as autoimmune hepatitis, based on normal total IgG level and absence of antismooth muscle antibodies. The ceruloplasmin level was 0.22 gr/l [N: 0.2–0.6]. No biliary tract disease or vascular alterations were detected on abdominal echography. Acute hepatitis due to WD was considered as the most likely diagnosis. Histological features observed at liver biopsy obtained by the transjugular route were consistent with the diagnosis, showing cirrhosis associated with chronic inflammatory changes, mild (10%) macrovesicular steatosis, and few ballooned hepatocytes. Increased hepatocyte copper-associated protein deposition was observed in periportal areas using Victoria blue stain (Figure 1). A quantification of 8071 *μ*g/gr in liver tissue [N: 10–35] was consistent with a massive accumulation of copper [1, 3]. Additional studies included a major increase in serum and urinary copper values of 2205 *μ*g/l [N: 70–175] and 4130 *μ*g/24 h [N: 15–60], respectively. A slit-lamp examination was inconclusive regarding Kayser–Fleischer's ring. Brain magnetic resonance imaging (MRI) was not performed at that time. Thus, according to the Leipzig score [3, 5], a diagnosis of AWH with cirrhosis and liver insufficiency was made, and a combined treatment of oral D-penicillamine (1200 mg/d) and zinc gluconate (90 mg/day) was promptly initiated on day 1 after admission. However, the following days were associated with a rapid clinical deterioration and majoration of hemolytic anemia with low hemoglobin levels in the range of 60 to 70 gr/l, necessitating repeated blood transfusions (Figure 2). At this stage, with a MELD score of 31, the patient did not reach Clichy criteria for urgent LT [6, 7] (no hepatic encephalopathy and a factor V level of 35%). Nevertheless, a WD prognostic index [8] of 11 indicated a poor prognosis with medical treatment alone. Therefore, considering the role of massive copper overload in the severe hemolysis coexistent with acute liver and renal failure (serum creatinine 177 *μ*mol/l [N: 44–80]), we decided to combine conventional chelation therapy with a mechanical method to rapidly remove excess serum copper. In spite of a very limited experience in the literature, a multidisciplinary team including hepatologists, nephrologists, and intensive-care specialists decided to use the Molecular Adsorbent Recirculating System (MARS) (MARS-Flux, Gambro, Lund, Sweden) as an albumin-based dialysis able to remove free exchangeable copper molecules in addition to other albumin-bound toxins associated with liver failure [9]. This technique has been preferred to therapeutic plasma exchange (TPE), reported to bring benefits as a bridge for liver transplantation in anecdotal reports [10, 11], because the use of fresh frozen plasma replacement would have influenced coagulopathy as a criterion to decide urgent listing [7].

**Figure 3:**
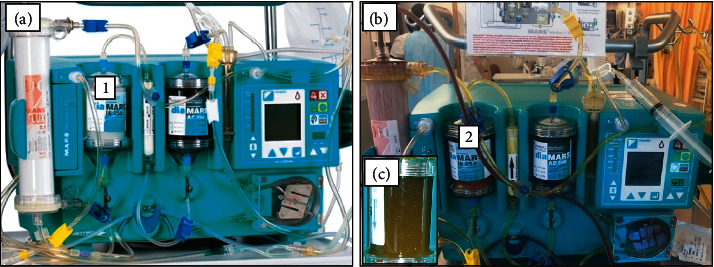
Illustration of the MARS device prior to use (a) and at the end of the initial session (b). The initially colorless diaMARS IE250 adsorber cartridge (1) became dark brown at the end of the procedure due to massive copper deposits (2). (c) A cartridge from a historical case of alcoholic cirrhosis and hepatorenal syndrome under MARS therapy showing, instead, a yellowish brown color due to bilirubin pigments.

**Figure 4:**
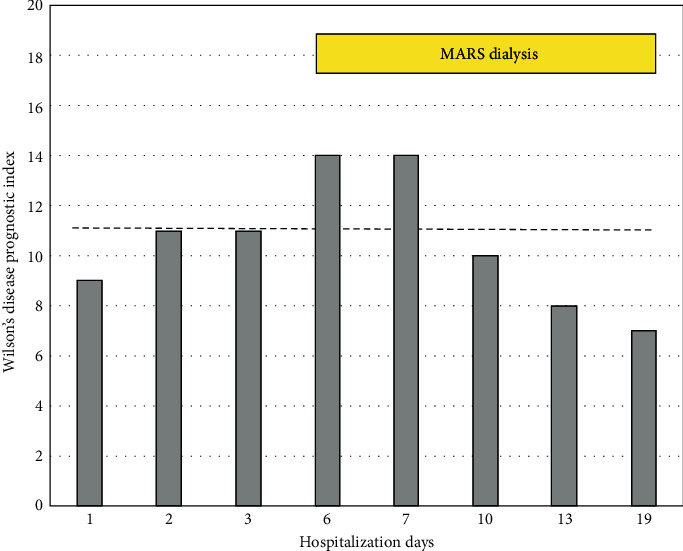
Evolution of Wilson's disease prognostic index during hospitalization. A score ≥ 11 (dashed line) is associated with a high probability of death without LT according to [8].

MARS is a hemodialysis system coupled to a closed circuit containing an albumin-rich dialysate with a carbon filter and an anion exchanger. The device aims to mimic the liver detoxification mechanisms at the hepatocyte membrane level. The plasma albumin loaded with toxins will, at the level of the MARS filter, transfer the albumin-bound toxins *via* a membrane permeable to toxins but not to proteins (cut-off 30 kDa) [12], therefore minimizing the depletion in hormones, proteins, and platelets. From a technical point of view, MARS therapy is coupled with hemodiafiltration using the MARS Prismaflex system which includes both one filter in contact with blood (MARS-FLUX) and one in contact with albumin. The MARS set also includes one adsorber column filled with carbon charcoal (diaMARS AC250) eliminating nonpolar compounds (i.e., fatty acids) and another column filled with ion-exchanger resin (diaMARS IE250) to eliminate anionic molecules including bilirubin and copper. The MARS system was administered to our patient using a 13F dialysis catheter in the femoral vein according to the manufacturer's recommendations adapted to our institution's guidelines. A total of 9 MARS dialysis sessions (each session lasting 6–8 hours) were administered starting from day 6 to day 17, with an excellent clinical tolerance, no bleeding complications, and only a marginal decrease in platelet count.  Figure 3 depicts the MARS device and the dark-brown-colored adsorber cartridge filled with copper. The patient rapidly recovered from hemolysis, as no further blood transfusions were necessary after day 7 (see Figure 2). The evolution of WD prognostic index during the hospitalization is depicted in Figure 4. On day 21, the MELD score was 14, hemoglobin level was 87 gr/l, LDH had decreased from 2004 U/L to 327 U/L [N: 87–210], and the patient's general condition was good. She left the hospital on a combined treatment of penicillamine 1200 mg/d and zinc gluconate 90 mg/d and received also dietary counseling. At the outpatient visit 3 weeks later, hemoglobin was 133 gr/l, and the urinary copper excretion value was reduced at 1080 *μ*g/24 h compared to baseline measure (−74%). Determination of serum copper level was not repeated thereafter. The brain MRI scan did not show T2 hyperintensity in basal ganglia.

## 3. Discussion

This is a typical clinical presentation of AWH with background chronic liver disease, presenting as an acute-on-chronic liver failure [13]. Wilson's disease is a rare autosomal recessive disorder of copper metabolism resulting in a progressive accumulation of copper in several organs including the liver and the brain [3]. Clinical presentation includes neurological symptoms, paucisymptomatic chronic liver disease, or acute liver injury [13] which accounts for approximately 10% of acute liver failure cases referred for urgent LT [14]. Laboratory values of AWH may show typical alterations including a low alkaline phosphatase and modest elevations of transaminases levels, while serum ceruloplasmin, the concentration of which is expected to be low, may be normal under the influence of inflammation. A Coombs-negative hemolytic anemia is suggestive of the diagnosis of acute WD [15]. High values of non-ceruloplasmin-bound copper and 24-hour cupruria are typical of AWH, but similar alterations may also be observed in acute hepatitis of other etiologies due to passive release of copper from hepatocytes. Thus, when interpretation is difficult, a liver biopsy with quantification of hepatic copper, as in our case, helps to make a final diagnosis.

Liver failure due to AWH is a clinical challenge. Once the diagnosis is established, it is crucial to determine early if pharmacological chelation therapy will be efficient enough to remove excess copper and progressively improve liver function [16] or if urgent liver transplantation is required [3]. On the one hand, according to a value of 11 in the New Wilson Prognostic Index published by Dhawan et al. [8], there was a high risk of death without transplantation. On the other hand, Clichy's criteria used in our institution for the management of fulminant hepatic failure were not met and, thus, the patient was not immediately listed for urgent LT. In spite of a rapid initiation of both the copper-chelating agent (penicillamine) and an intestinal absorption copper blocker (zinc gluconate), biological tests continued to deteriorate with renal and hematological complications. It was considered that massive copper overload was primarily responsible for both the development of acute tubular injury with renal failure and the severe intravascular hemolysis [17] requiring repeated blood transfusions.

The extracorporeal detoxification methods that are available to rapidly remove excess copper in acute WD include TPE and MARS dialysis. According to recent American guidelines on apheresis, TPE is the first-line therapy as a bridge to liver transplantation in WD, although the recommendation level is low [18]. TPE removes both ceruloplasmin and albumin-bound copper, improves coagulation parameters due to concomitant plasma administration, is available in most healthcare settings, but uses citrate which is not recommended in acute liver failure. The MARS system combines a separate albumin circuit and two columns including a large ion-exchanger resin cartridge that can be used until saturation and uses unfractionated heparin during the procedure. These technical characteristics make this particular extracorporeal detoxification device a suitable option in this clinical situation [19–21]. In addition, conventional dialysis combined with MARS clears water-soluble substances such as creatinine, urea, and ammonium that participate in hepatic encephalopathy and hepatorenal syndrome [22].

In our patient, repeated MARS dialysis combined with conventional medical treatment allowed a rapid control of hemolysis, as blood transfusions were no longer necessary after the second MARS session. This improvement was associated with a progressive decrease in LDH level as a marker of hemolysis [4].

The survival benefit of MARS in acute-on-chronic liver failure is not proven [23]. Based on few case series [5, 20, 24] and clinical practice guidelines on WD from India [25], MARS dialysis could stabilize the patient's condition and may serve as a bridge to LT. Our observation demonstrates that MARS sessions administered to target massive copper-overload-associated hemolysis reduced the need for repeated blood transfusions and have contributed to the patient's survival without urgent LT.

## Data Availability

No data were used to support this study.

## Abbreviations

Hb: Hemoglobin
INR: International Normalized Ratio
WD: Wilson's disease
AWH: Acute Wilsonian hepatitis
MARS: Molecular adsorbents recirculating system
TPE: Therapeutic plasma exchange
MELD: Model for end-stage liver disease
LT: Liver transplantation.
